# A qualitative study of workplace violence among healthcare providers in emergency departments in India

**DOI:** 10.1186/s12245-020-00290-0

**Published:** 2020-06-17

**Authors:** Kevin Davey, Veda Ravishankar, Nikita Mehta, Tania Ahluwalia, Janice Blanchard, Jeffrey Smith, Katherine Douglass

**Affiliations:** 1 Department of Emergency Medicine, George Washington University, 2120 L Street NW, Suite 450, Washington, DC 20037 USA; 2 Children’s National Medical Center, Washington, DC, USA

**Keywords:** Healthcare workplace violence, Violence against healthcare workers, Developing emergency care systems, India

## Abstract

**Background:**

Emergency department (ED) workplace violence is increasingly recognized as an important issue for ED providers. Most studies have occurred in developed countries with established laws and repercussions for violence against healthcare providers. There is a paucity of data on workplace violence against ED providers in less developed countries. The aim of this study was to learn more about workplace violence among healthcare providers in EDs in India.

**Results:**

Semi-structured interviews were conducted in-person with physicians, nurses, and paramedics in Indian EDs. Interviews were coded independently using the NVivo qualitative research software. A hybrid thematic analysis approach was used to determine dominant themes. Sixty-three interviews were conducted at 7 sites across India. Interview participants include attending physicians (11), resident physicians (36), nurses (10), and paramedics (5). Events were most often described as involving accompanying persons to the patient, not the patient themselves. Most events involved verbal abuse, although a significant percentage of responses described some kind of physical violence. ED factors such as busy times with high patient volumes or periods of waiting are associated with increased violence, as well as incidents with unanticipated outcomes such as patients with severe illness or death. Decreased levels of health literacy among patients often contribute as the financial stressors of paying for medical care. Providers reported negative consequences of workplace violence on quality of care for patients and their own motivation to work in the ED. Communication strategies were frequently proposed as interventions to mitigate violence in the future including both provider communication as well as public awareness campaigns.

**Conclusion:**

Workplace violence is a frequent reality for this sample of Indian ED healthcare providers. Alarming levels of verbal and physical abuse and their impact on patient care are described. This qualitative study identified unique challenges to Indian ED providers that differ from those in more developed settings, including financial stressors, inadequate enforcement of rules governing behavior in the hospital, and an overwhelming frequency of violence emanating from patient family members and attendants rather than the patients themselves. Further investigation into preventive strategies is needed.

## Background

Previous studies have documented the prevalence of workplace violence against healthcare providers [1–4]. The ED is particularly prone to violence given the multitude of stressors present in the emergency setting including, but not limited to, high patient volume, high acuity of patient illness, rotating staff, and late hours [5–7]. While prior studies have examined the phenomenon of violence against emergency healthcare providers, the vast majority of these studies have been conducted in developed western countries with established laws and legal repercussions for violence against healthcare providers. Few studies have been conducted in low resources settings where regulations protecting healthcare providers are more sparse [8]. Likewise, in many low resource settings, the field of emergency medicine (EM) is still in its infancy and patient understanding of what care is available in the emergency setting may be lacking, potentially further exacerbating the risk of violence. Of those studies that have been conducted in India and other low resource settings, most have been limited in scope, either focusing exclusively on one type of provider (physicians vs nurses vs paramedics), or limited to a single institution [9–11].

In order to address the issues of violence against emergency healthcare providers in India and other developing emergency care systems, a greater understanding of the unique issues surrounding violence in EDs in low-resource settings is needed. The objective of this study is to gain a better understanding of issues surrounding violence against healthcare providers in Indian ED’s. To our knowledge, this represents the first multicenter, qualitative study of workplace violence among ED healthcare providers in India.

## Methods

**Fig. 1:**
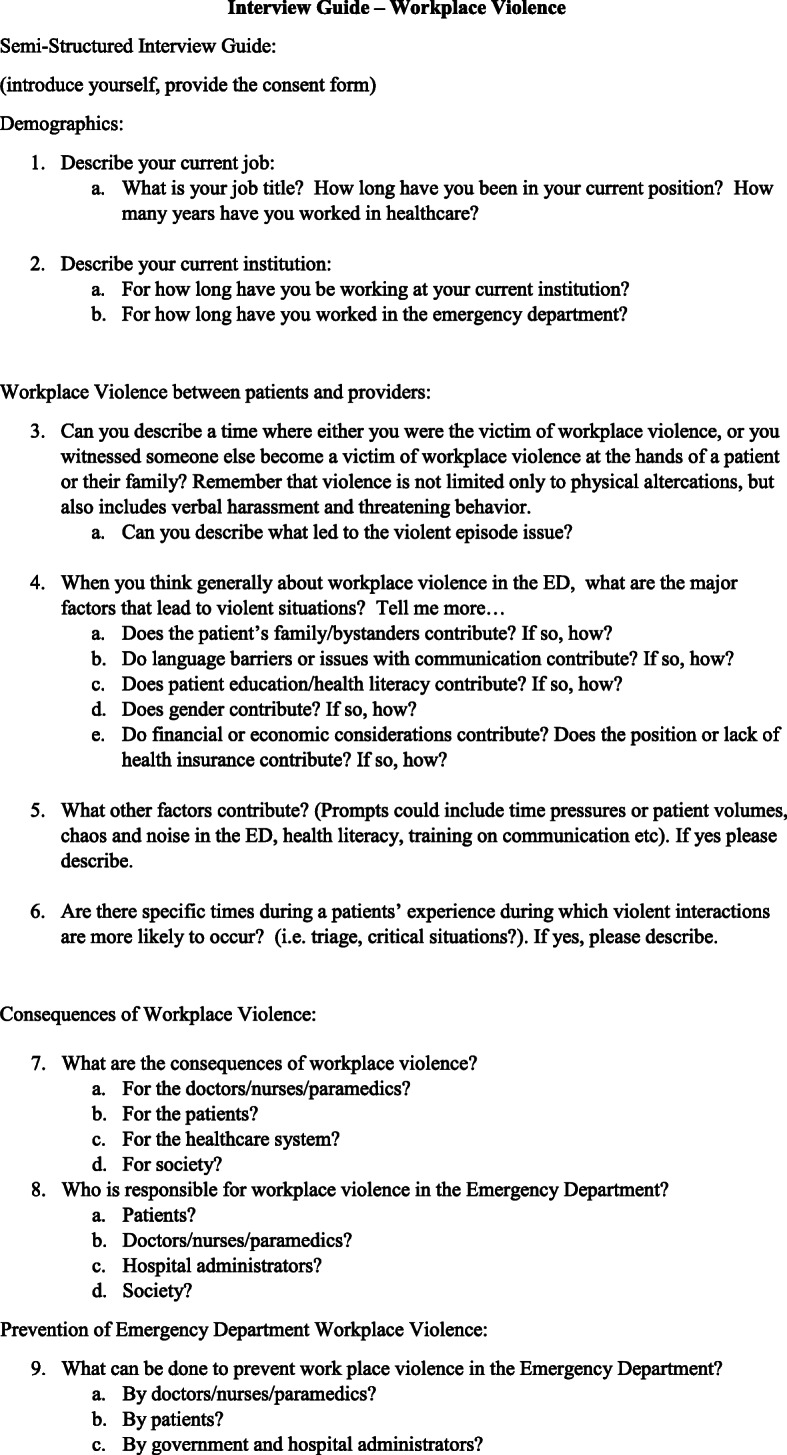
Interview guide—workplace violence

Semi-structured interviews were conducted in person with attending emergency physicians, emergency medicine residents, nurses and paramedics at seven EDs across India. Interviews were conducted by student field researchers who underwent training in proper interview techniques prior to beginning the study. The semi-structured interview guide was developed based on prior studies of violence against healthcare providers and was piloted before initiating the study (Fig. 1). The study was approved by the George Washington University Institutional Review Board and verbal consent was obtained from all interview participants. The interviews were conducted in English in a private hospital conference room without interruption. With the exception of the participants’ job title (i.e., physician, nurse, paramedic), no personal identifying information was collected. Interviews were recorded and transcribed.

A hybrid thematic analysis approach was used to determine dominant themes. Interviews were coded independently by two blinded researchers using the NVivo qualitative research software. The independently coded interviews were merged and discrepancies were addressed by a third researcher. The coding scheme can be found in Table 1. The design and reporting of data were based on the consolidated criteria for reporting qualitative research (COREQ) guidelines.

**Table 1:** Coding scheme

Theme	Sub-themes
Types of violence	Verbal violence, physical violence
Experiences of violence	Between patients and doctors, between attendants/bystanders and doctors, between patients and nurses, between bystanders and nurses, between patients and other hospital staff, between bystanders and other hospital staff
Causes of violence	Financial, health literacy, communication challenges, emotional factors, perpetuation based on media/prior events, gender factors, ED factors (crowding, noisiness, long wait times, etc), age factors, intoxication
Description of events	Timing, involving physicians, involving nurses, involving other ED staff, involving patients, involving family or attendants/bystanders
Consequences	On doctors or providers, on patients and their care, on society
Responsibility	Patients, bystanders/attendants, nurses, doctors, other hospital staff, hospital, society
Prevention strategies	Improved communication, improved knowledge and skills (among physicians/providers), improved patient and societal education, improved security, hospital interventions, government interventions

## Results

**Fig. 2:**
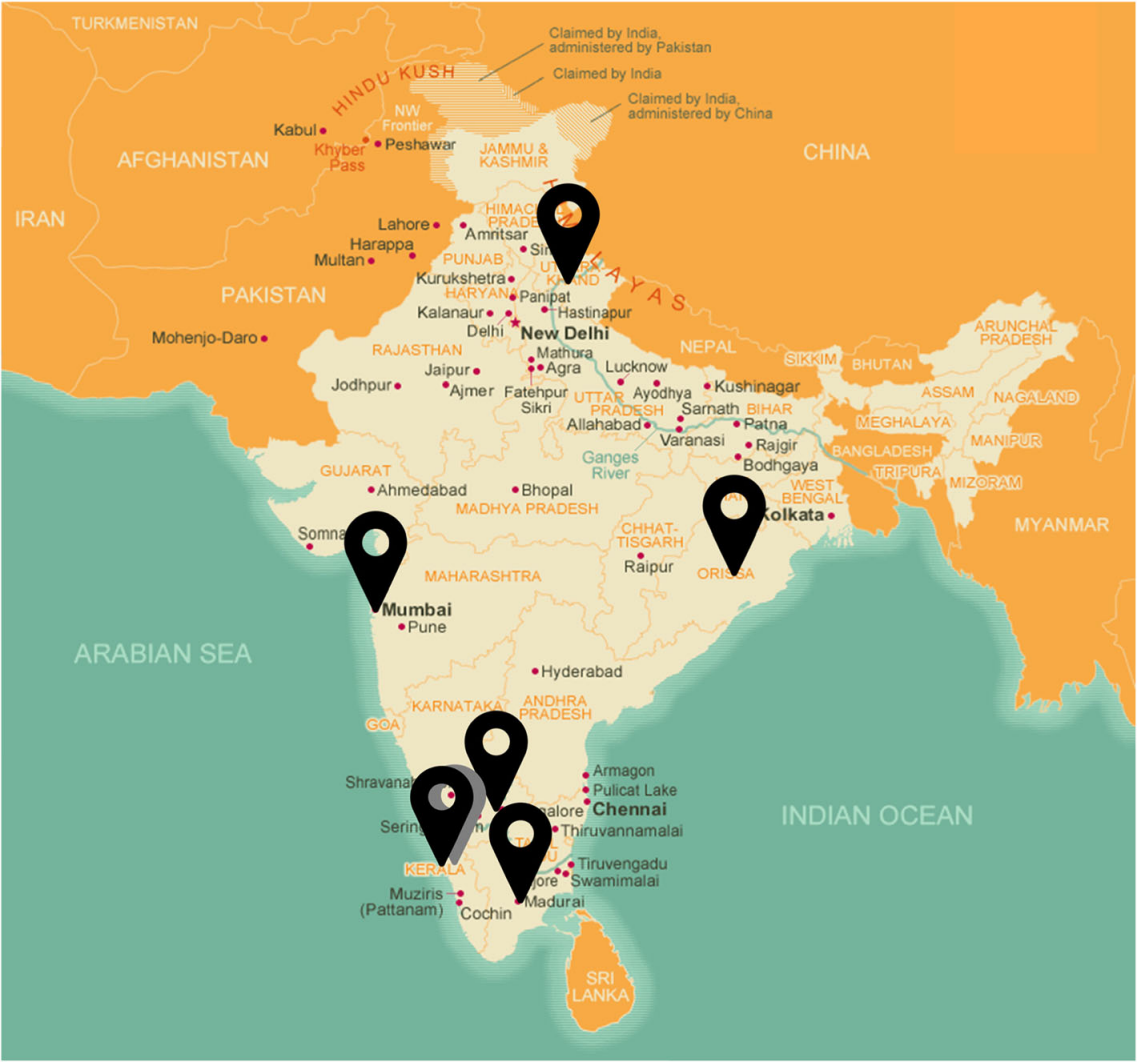
Map of hospital locations

Sixty-three interviews were conducted at 7 hospital ED’s across India. A map of hospital locations can be found in Fig. 2. Interviewees include 11 attending physicians, 36 resident physicians, 10 nurses, and 5 paramedics. One interview participant did not give their job title. Common themes were described from the coding scheme and frequencies and relative frequencies of each subtheme are represented in Table 2. Representative quotations from each theme can be found in Table 3. Seven themes emerged from thematic analysis: types of violence, experiences of violence, causes of violence, description of violence events, consequences of violence, responsibility for the violence, and prevention strategies. Detailed discussions of each theme can be found below.

**Table 2:** Response frequencies and relative frequencies

**Types of violence**	**Frequencies** (*n* = 205)	**Relative frequency**
Sub-theme: verbal	167	81.4%
Sub-theme: physical	38	18.5%
**Experiences of violence**	**Frequencies** (*n* = 126)	**Relative frequency**
Sub-theme: between attendants/bystanders and doctors	69	54.8%
Sub-theme: between attendants/bystanders and nurses	16	12.7%
Sub-theme: between attendants/bystanders and other hospital staff	16	12.7%
Sub-theme: between patients and doctors	15	11.9%
Sub-theme: between patients and nurses	4	3.2%
Sub-theme: between patients and other hospital staff	6	4.8%
**Causes of violence**	**Frequencies** (*n* = 686)	**Relative frequency**
Sub-theme: financial	91	13.2%
Sub-theme: health Literacy	162	23.6%
Sub-theme: communication challenges	80	11.7%
Sub-theme: emotional factors	73	10.6%
Sub-theme: gender factors	26	3.8%
Sub-theme: perpetuation based on media/prior events	9	1.3%
Sub-theme: age factors	31	4.5%
Sub-theme: intoxication	23	3.4%
**Description of events**	**Frequencies** (*n* = 346)	**Relative frequency**
Sub-theme: Timing	52	15%
Sub-theme: involving physicians	44	12.7%
Sub-theme: involving nurses	23	6.6%
Sub-theme: involving other ED staff	8	2.3%
Sub-theme: involving patients	42	12.1%
Sub-theme: involving family or bystanders	177	51.2%
**Consequences of violence**	**Frequencies** (*n* = 190)	**Relative frequency**
Sub-theme: on doctors or providers	113	59.5%
Sub-theme: on patients and their care	57	30%
Sub-theme: on society	20	10.5%
**Responsibility for violence**	**Frequencies** (*n* = 131)	**Relative frequency**
Sub-theme: patients	21	16%
Sub-theme: bystanders/attendants	23	17.6%
Sub-theme: nurses	4	3.1%
Sub-theme: doctors	19	14.5%
Sub-theme: other hospital staff	9	6.9%
Sub-theme: hospitals	22	16.8%
Sub-theme: society	33	25.2%
**Prevention strategies**	**Frequencies** (*n* = 285)	**Relative frequency**
Sub-theme: improved communication	79	27.7%
Sub-theme: improved knowledge and skills (among physicians/providers)	13	4.6%
Sub-theme: improved patient and societal education	57	20%
Sub-theme: improved security	45	15.8%
Sub-theme: hospital interventions	75	26.3%
Sub-theme: government interventions	16	5.6%

**Table 3:** Representative quotations

Theme	Quotation
Types of violence	“Verbal, almost on a daily basis, especially when it gets busy. Recently an attender, because we didn’t have beds, was very frustrated. He started calling us words like ‘idiots, fools.’”
Experiences of violence	In regards to a recent patient who presented with abdominal pain and difficulty breathing who later went into septic shock, “They started shouting, ‘What have you done with our patient? How did she become hypotensive? Initially her BP was fine!’ And they were not able to understand… They were talking like, ‘You didn’t give her anything to drink so that’s why she went into shock!’ Two or three attendants were there and all of the were shouting and the situation escalated quite a bit.”
Causes of violence	“People shouting, demanding for fast disposition in spite of other patients. When you are attending some other serious patient, they don’t understand the fact that the other patient needs much more attention. They just want their part to be cleared off and to be sent home.”
Description of events	“One patient arrested, so my colleague immediately started CPR. But when the patient attender came inside, they thought we were beating him… The lady, I think the patient’s wife, she gave a slap to my colleagues… it took so long to convince them what actually happened.”
Consequences	“For the residents and physicians, it creates and unhealthy environment and a lack of trust.”
Responsibility	“It’s not only patient bystanders. If any violence is happening in the ED we can combat the violence by constantly counseling the patient attenders regarding the process what’s going on. Suppose a patient comes and we say ‘this lab takes 2-3 h, this is the cost.’ If we counsel everything from this point to point the violence part at least will calm down. See India is a developing country, perception is different actually. What the general public thinks is this hospital charge more, they work like a corporate sector, they don’t bother about patients, they bother only about the money.”
Prevention strategies	“When we are receiving patients inside our ER we have to make the attenders await outside. If we avoid attenders entering our department, we can avoid violence.”

### Types of violence

Interview participants described the types of violence they experienced in the ED. The most commonly reported type of violence against healthcare providers was verbal abuse (81.4%), although a significant percentage of respondents also reported experience with physical abuse (18.5%). Participants reported almost daily episodes of being shouted at or degraded by patients and their family members or attendants, as well as several who described episodes in which they or their colleagues were physically assaulted.

### Experiences of violence

Interview participants described their personal experiences with workplace violence. Over 90% of interview participants (*n* = 57) reported personal experience with workplace violence. The most common descriptions were between a patient’s family members or attendants and doctors (54.8%), nurses (12.7%), and other hospital staff (12.7%). Overall, family members or attendants were identified as the most common perpetrators of violence against ED staff. Reported frequencies of violence by patients’ family members or attendants outnumbered violence by patients by more than 4:1. As one resident put it, “It’s mostly the bystanders, and the more number of bystanders, the more likely.” [sub-theme: between attendants/bystanders and doctors].

### Causes of violence

Interview participants described what they perceive to be the most common precipitants of workplace violence in the ED. ED dependent factors, such as crowding or wait times, were reported as the most common precipitant of violence against ED providers (27.8%), followed by a lack of health literacy among patients (23.6%), and patients’ financial concerns (13.2%). Interviewees identified that many patients are unfamiliar with the idea of an acuity based triage system, and expect to be seen immediately when they present to the ED. As lower acuity patients are forced to wait they are more likely to become agitated and engage in violence. As one participant described, “The patients don’t understand. In western countries the expected waiting time is 3-4 h. That’s not the case in India. If you ask the patient to wait hardly 10-15 min or 30 min, things go berserk.” [sub-theme: health literacy]. Interview participants also identified the importance that financial implications can play in precipitating workplace violence in the ED. These issues may be exacerbated at private hospitals which may charge more for services. As one participant described, “See India is a developing country, perception is actually different here. What the general public thinks is this hospital charges more, they work like the corporate sector, they don’t bother about patients, they bother only about money.” [sub-theme: financial issues].

Interviewees report that a lack of basic health literacy among patients may serve to exacerbate violence in the ED. As one participant stated “People come with lots of expectations, and the patients are at such critical conditions or at the end stage, that they expect from us that we’ll do some magic. That within a few minutes or an hour or so, their patient will be in better condition. But when eventually that doesn’t happen or the condition of the patient deteriorates they don’t accept it.” [sub-theme: health literacy]. Other common precipitating factors identified by interviewees included emotional factors, such as unanticipated patient outcomes or death (10.6%), communication challenges (11.7%), factors relating to the patient’s age (4.5%), and factors relating to the gender of the patient or the provider (3.8%). Participants stated that violence was more likely to occur with a pediatric patient. As one interview participant described, “If the patient is a child then the parent tends to get angry fast.” [sub-theme: age factors]. Interview participants also observed that gender factors may play a role in ED violence. As one participant noted, “Sometimes the relatives don’t think, seeing a female doctor, that she is confident or she is good enough to treat the patients… They are okay getting treated by a first year who is a male, but not a female doctor who in her second or final year.” [sub-theme: gender factors]. Participants noted that intoxication may play a role in ED violence (3.4%); however, it was identified with relatively little frequency as compared with other common precipitants.

### Description of events

Interview participants were asked to describe the circumstances surrounding their experiences with workplace violence in the ED. Common themes that emerged were the involvement of family members or attendants in violent events (51.2%) and the role that timing may play in precipitating events (15%). Many interview participants noted that violent events were more likely to take place at night, when lower ED staffing may result in longer wait times. Furthermore, many interviewees stated that hospital security staffing is also decreased during night shifts and perpetrators of violence against ED providers may feel emboldened by the lack of security personnel.

### Consequences

Interview participants were asked to identify the consequences of workplace violence in the ED. Participants overwhelmingly believed that healthcare providers suffered the greatest adverse consequences of ED violence (59.5%), but also identified patients and patient care (30%) as well as society at large (10.5%) as suffering adverse consequences. Interview participants reported a decrease in morale as a result of violent events and acknowledged that fear of violence may affect their medical decision-making, causing them to treat patients in the manner least likely to result in a violent outcome, rather than doing what is medically indicated. As one resident described “Definitely it will affect our work pattern and work efficiency, because we will have to concentrate more time on solving that issue rather than taking care of patients. Other patients will be affected and they will be kept unattended so that also becomes a problem.” [sub-theme: on doctors and providers].

### Responsibility

Interview participants were asked to identify whom they believe were responsible for workplace violence in the ED. A variety of different groups were identified including society (25.2%), patient family members or attendants (17.6%), hospitals (16.8%), patients (16%), and doctors (14.5%). Participants acknowledged that much of the violence against healthcare providers in the ED may result from misunderstandings and distrust between patients, their family members, and providers. Participants believe that the root of this misunderstanding is multifactorial, resulting from a lack of basic medical education among the general ED population as well as poor communication between providers and their patients. Many participants also blamed hospitals for failing to provide adequate security as well as failure to enforce hospital rules limiting the number of patient attendants allowed in the ED.

### Prevention strategies

Interview participants were asked to offer prevention strategies to mitigate violence against ED providers. Proposed strategies included improved communication between healthcare providers and patients (27.7%), hospital-based interventions (26.3%), and improved patient and society education (20%). Improved communication between patients, their attendants, and providers was repeatedly suggested as a way to decrease incidence of violence in the ED. Participants also called on hospitals to improve security, suggesting measures like increasing the number of security personnel in the ED, adding hospital metal detectors, and greater enforcement of hospital rules on the number of attendants allowed in the ED. Public outreach to improve the medical knowledge of the general population was also seen as an important step. Participants also called for greater government enforcement of laws prohibiting violence against healthcare providers.

## Discussion

Violence against ED healthcare providers is an unfortunate yet common occurrence in Indian EDs. Over 90% of ED healthcare providers interviewed described personal experience with workplace violence. This is consistent with prior studies conducted in more developed countries, which have found a prevalence of violence against ED healthcare providers between 80% and 100% [5, 12–14]. With the caveat that existing ED workplace violence data in India is extremely limited, the prevalence found in this study is somewhat higher than previously reported. In the only prior published study to address the issue, a survey of ED residents at a single institution, 89% of respondents said they had witnessed some form of workplace violence, but only 70% reported having been the victim of violence themselves [9]. The reason for this difference is difficult to ascertain given the dearth of data, but highlights the need for continued research into the issues surrounding ED workplace violence in India.

Several common patterns of violence emerged throughout the course of these interviews. While most of the reported violence is verbal abuse, almost one in five providers reported experience with physical abuse. This is also consistent with prior studies conducted in higher resource settings, which demonstrate similar incidents of both verbal and physical abuse [5, 13, 14]. The commonalities between the patterns of workplace violence in India as compared with higher resource settings can likely be attributed to factors that make the ED especially prone to violence in general. Long wait times, high patient volumes, rotating staff, and a high acuity of illness have all been linked to increases in workplace violence in the ED in more developed countries [14, 15]. In our study, interview participants cited ED related factors, such as wait times and unexpected patient outcomes, as the most frequent precipitants of violence against ED providers. Low health literacy among patients was also identified as a common precipitant of violence, similar to findings from prior studies in higher resource settings [14, 15].

The findings from these interviews differ from those conducted in higher resource settings in several important ways. One precipitant of violence frequently cited by interview participants that differs substantially from more developed countries was financial concerns. While access to health insurance is increasingly common in India, many people must still pay out of pocket for healthcare expenses [16]. Unlike in more developed countries, where bills for hospital expenses arrive weeks to months later, in India many patients are presented with a bill at the time of their visit and required to pay before leaving the hospital. In a prior qualitative study conducted at a single hospital in central India, doctors identified growing distrust in the doctor-patient relationship, which they in part attributed to “high payments and high expectations.” In the study, doctors stated that a combination of increasing denial of care for non-paying patients, as well as a refusal to accept poor outcomes by the “buyer of costly healthcare,” were eroding the fundamental building blocks of the doctor-patient relationship [10]. These observations are consistent among responses of several interview participants who repeatedly cited experiences of violence around unanticipated poor outcomes, and family members upset by the cost of care. Findings from studies in other low-income settings also cite a growing public distrust of medical professionals and the perception that doctors and hospitals are abusing their positions, accepting bribes and providing lower-quality care to poorer patients [17]. Couple this strained sense of trust with the already fraught ED environment, and it is easy to envision how adding financial stressors, like presenting a patient with a bill, may serve to exacerbated ED workplace violence.

Another major difference reported between ED violence in India and that of more developed countries is the perpetrators of violence. Interview participants overwhelmingly identified those who accompany the patient to the ED, commonly referred to as bystanders or attendants, rather than the patients themselves, as the main perpetrators of violence against ED healthcare providers. This stands in stark contrast to other studies in more developed countries, which have shown that patients are directly involved in up to 90% of all violent events, while friends or family members are implicated in only 11% of violent episodes [5]. Likewise, while in more developed settings, the vast majority of violent perpetrators have dementia, decompensated mental illness, or intoxication, our interview participants only rarely cited the presence of these factors [14, 18]. The reasons for this distinction may be a result of both cultural and regulatory differences. In India, it is extremely uncommon for a person to present to a healthcare setting unaccompanied by a friend or family member. In fact, many hospitals require that patients have an attendant with them in order to be admitted [19]. These attendants may act as a patient advocate, and even assist with minor nursing duties like basic hygiene and medication administration. While some hospitals have rules limiting the number of attendants, our interview participants report that these rules are rarely enforced. As one participant stated “If we receive a trauma patient, like 10 to 15 attenders will be there. All attenders will be very aggressive.” [theme: description of events; sub-theme: involving bystanders]. Violence perpetrated by patient attendants is also complicated by the tools available to mitigate that violence. While intoxicated or mentally unstable patients can be medicated, violent family members frequently cannot.

Interview participants frequently faulted a lack of adequate security for exacerbating violence in the ED. Participants also reported an increase in violent incidents at night, when there is less security available and when it may be slower to respond. These findings have not been previously described in India, though they are similar to findings from other studies in low resource settings, where providers cited a lack of confidence in the capacity and ability of security staff to provide a safe environment [20].

Providers reported negative consequences of workplace violence on the quality of care for patients and their own motivation to work in the ED. These findings match those from prior studies in higher resource settings, which link ED workplace violence to increased rates of burnout, missed workdays, and job dissatisfaction [5, 7, 21].

Solving the problem of workplace violence will require a multifaceted approach, addressing the perpetrators of violence, the considerations of healthcare workers, government intervention, law enforcement, and healthcare organizations. Interview participants identified a number of strategies for mitigating the incidence of violence against ED providers. Improved communication strategies, as well as public outreach campaigns to improve societal education and awareness, were frequently proposed interventions. While many strategies have been proposed for combating ED workplace violence in higher resource settings, few have any supporting evidence. The use of filmed vignettes as well as de-escalation training for healthcare workers has been proposed, but evidence for their efficacy is currently lacking [17, 22]. Hospital -based interventions like increased security, the use of hospital metal detectors, and better enforcement of laws limiting the number of attendants allowed in the ED were also frequently proposed in this study. While the increased use of metal detectors has been shown to aid in confiscation of dangerous weapons, there is little evidence to suggest that it actually decreases the incidence of violence [23]. Ultimately, decreasing workplace violence against ED providers will require a combined approach that utilizes multiple strategies and is customized to unique aspects of each environment.

## Limitations

This study was completed in private hospitals. Private hospitals in India may charge higher rates than public hospitals and thereby may exacerbate the role of financial factors in precipitating workplace violence in the ED. Additionally, the majority of interview participants were doctors, either residents or attendings/consultants. There may be a selection bias that the responses given overly represent the points of view and issues of physicians.

## Conclusion

Workplace violence is an unacceptable, although frequent, reality for ED physicians and staff in India, with alarming rates of verbal and physical abuse. This qualitative study identified unique challenges to Indian ED providers that differ from those in more developed settings, including financial stressors, inadequate enforcement of rules governing behavior in the hospital, and an overwhelming frequency of violence emanating from patient family members and attendants rather than the patients themselves. Further investigation into preventive strategies must be a priority.

## Data Availability

The datasets used and/or analyzed during the current study are available from the corresponding author on reasonable request.

## Abbreviations

ED: Emergency department
EM: Emergency medicine
